# Co-infections with multiple genotypes of *Anaplasma marginale* in cattle indicate pathogen diversity

**DOI:** 10.1186/s13071-017-2595-5

**Published:** 2018-01-03

**Authors:** Paidashe Hove, Mamohale E. Chaisi, Kelly A. Brayton, Hamilton Ganesan, Helen N. Catanese, Moses S. Mtshali, Awelani M. Mutshembele, Marinda C. Oosthuizen, Nicola E. Collins

**Affiliations:** 1 Vectors and Vector-borne Diseases Research Programme, Department of Veterinary Tropical Diseases, Faculty of Veterinary Science, University of Pretoria, Pretoria, South Africa; 2 Biotechnology Platform, Agricultural Research Council, Onderstepoort, Pretoria, South Africa; 3 Research and Scientific Services Department, National Zoological Gardens of South Africa, Pretoria, South Africa; 4 Department of Veterinary Microbiology and Pathology, Washington State University, Pullman, WA USA; 5 Inqaba Biotechnical Industries, Hatfield, Pretoria, South Africa; 6 School of Electrical Engineering and Computer Science, Washington State University, Pullman, WA USA; 7 Present Address: National Research Foundation, Brummeria, Pretoria, South Africa; 8 Present Address: Forestry and Agricultural Biotechnology Institute, University of Pretoria, Pretoria, South Africa

**Keywords:** *msp1α*, *msp1β*, *groEL*, qPCR, Next-generation amplicon sequencing

## Abstract

**Background:**

Only a few studies have examined the presence of *Anaplasma marginale* and *Anaplasma centrale* in South Africa, and no studies have comprehensively examined these species across the whole country. To undertake this country-wide study we adapted a duplex quantitative real-time PCR (qPCR) assay for use in South Africa but found that one of the genes on which the assay was based was variable. Therefore, we sequenced a variety of field samples and tested the assay on the variants detected. We used the assay to screen 517 cattle samples sourced from all nine provinces of South Africa, and subsequently examined *A. marginale* positive samples for *msp1α* genotype to gauge strain diversity.

**Results:**

Although the *A. marginale msp1β* gene is variable, the qPCR functions at an acceptable efficiency. The *A. centrale groEL* gene was not variable within the qPCR assay region. Of the cattle samples screened using the assay, 57% and 17% were found to be positive for *A. marginale* and *A. centrale*, respectively. Approximately 15% of the cattle were co-infected. *Msp1α* genotyping revealed 36 novel repeat sequences. Together with data from previous studies, we analysed the Msp1a repeats from South Africa where a total of 99 repeats have been described that can be attributed to 190 *msp1α* genotypes. While 22% of these repeats are also found in other countries, only two South African genotypes are also found in other countries; otherwise, the genotypes are unique to South Africa.

**Conclusions:**

*Anaplasma marginale* was prevalent in the Western Cape, KwaZulu-Natal and Mpumalanga and absent in the Northern Cape. *Anaplasma centrale* was prevalent in the Western Cape and KwaZulu-Natal and absent in the Northern Cape and Eastern Cape. None of the cattle in the study were known to be vaccinated with *A. centrale*, so finding positive cattle indicates that this organism appears to be naturally circulating in cattle. A diverse population of *A. marginale* strains are found in South Africa, with some *msp1α* genotypes widely distributed across the country, and others appearing only once in one province. This diversity should be taken into account in future vaccine development studies.

## Background

Bovine anaplasmosis is one of the most economically important tick-borne diseases of ruminants the world over [1–3]. The causative agent of the disease is the rickettsia *Anaplasma marginale,* a gram-negative, obligate intra-erythrocytic pathogen of the order Rickettsiales and family *Anaplasmataceae* [2, 4–6]. *Anaplasma marginale* is the most prevalent vector-borne pathogen and is found on all six inhabited continents [5, 7–9]. Approximately 20 tick species worldwide have been implicated as biological vectors of the pathogen, although mechanical and transplacental transmission has also been reported [2, 3, 10–15]. *Anaplasma centrale*, considered by some authors to be a subspecies of *A. marginale*, generally causes a milder, less virulent form of the disease, with occasional clinical cases [16]. Infection with *A. centrale* confers immunity to *A. marginale*. *Anaplasma centrale* has therefore been employed as a live vaccine [2, 17]. In South Africa, bovine anaplasmosis is found in most of the cattle farming regions and is an economically important tick-borne disease [2, 3, 17]. It is endemic in eight of the nine provinces of the country [3], except the Northern Cape where the tick vectors are absent. Five tick species, namely *Rhipicephalus decoloratus*, *R. microplus*, *R. evertsi evertsi*, *R. simus* and *Hyalomma marginatum rufipes,* have been shown experimentally to be capable of transmitting *A. marginale* in South Africa [12].

Recently we compared three nucleic acid-based tests for detecting *A. marginale* and *A. centrale* [18]. The nested polymerase chain reaction (nPCR) assay (which targets the *msp1β* gene of *A. marginale* and *msp2* of *A. centrale* [19, 20]) detected fewer *A. marginale* positive samples than the duplex quantitative real-time PCR (qPCR) (which detects *msp1β* of *A. marginale* and *groEL* of *A. centrale* [20, 21]). This discrepancy was found to be due to sequence variation in the *msp1β* gene in the target region of one of the internal PCR primers. The reverse line blot (RLB) hybridization assay [22], in which species-specific sequences in the 16S rRNA gene of *Anaplasma* and *Ehrlichia* species are detected, was found to be less sensitive than the qPCR and nPCR assays. The qPCR assay was thus shown to be the most appropriate assay for detection of *A. marginale* in blood samples from cattle [18]. However, the identification of *msp1β* gene sequence variants indicates the need to assess sequence variation in the target regions of the qPCR assays, to ensure that all *A. marginale* and *A. centrale* genetic variants are detected.

A genotyping method based on the *msp1α* gene [23–26], which encodes major surface protein 1a (Msp1a) [27, 28], has been developed for characterizing *A. marginale* strains in positive samples and has been applied throughout the world. *Anaplasma marginale msp1α* genotyping is not only useful for understanding the genetic diversity of the pathogen but has also been used to elucidate host-pathogen interactions and co-evolution [8, 25, 29–32]. *Msp1α* genotyping relies on variation in tandem repeats at the 5′ end of the gene that varies both in number and sequence. Msp1a repeats are identified in the deduced amino acid sequence and are given alphanumeric names to distinguish between sequence variants; the Msp1a repeat structure determines the *msp1α* genotype of a strain. Over 250 Msp1a repeats have been described, making it a useful marker for discriminating *A. marginale* strains [24–26, 28, 31, 33, 34]. In the South African context, *msp1α*-based genotyping has revealed diversity in *A. marginale* strains across the country, and novel repeats have been identified, although other repeats are identical to those detected in Europe and the USA [24, 25]. Although infection exclusion was thought to result in only one *A. marginale* genotype in individual cattle and ticks [35], more recently, infections with multiple distinct *msp1α* and *msp2* genotypes have been identified in herds in endemic areas with high infection rates [36–40].

In this study, we used next-generation amplicon sequencing to assess the level of variation in the qPCR target regions of the *msp1β* (*A. marginale*) and *groEL* (*A. centrale*) genes from field samples in order to ensure that the duplex qPCR assay [20, 21] was able to detect all *A. centrale* and *A. marginale* genetic variants in South Africa. The assay was then used to screen cattle samples from all nine provinces of the country for the presence of these organisms, followed by *msp1α* genotyping from selected positive samples. We cloned *msp1α* PCR amplicons and sequenced multiple clones to maximize the diversity of *A. marginale* genotypes detected from individual animals.

## Methods

### Blood sample collection and genomic DNA extraction

A total of 517 EDTA blood samples were obtained from mixed breeds of cattle from all nine provinces of South Africa (Table 1). These consisted of fresh blood samples collected from cattle in the Mnisi communal area (79) and a private farm near Lydenburg (17), Mpumalanga Province, and 148 samples collected from cattle at the University of Pretoria Experimental Farm (Proefplaas, Gauteng Province), as well as 284 frozen cattle blood samples, collected from different parts of South Africa, obtained from the National Zoological Gardens (NZG), Pretoria, South Africa. Blood samples from cattle were collected according to the animal ethics code of the University of Pretoria in 9 ml Vacuette® EDTA tubes (Greiner Bio-One, Kremsmünster, Austria), from the coccygeal vein of cattle that were at least 1 year old. *Anaplasma centrale* blood vaccine was obtained from Onderstepoort Biological Products (Pretoria, South Africa). Genomic DNA was extracted using the QIAamp DNA Blood Mini Kit (Qiagen, Hilden, Germany) according to the manufacturer’s instructions, and DNA was eluted in 100 μl elution buffer and stored at -20 °C.

**Table 1:** Number and origin of cattle field samples used in the study

Province	No. of samples
Limpopo	30
Mpumalanga	115
Gauteng	183
North West	30
Free State	30
KwaZulu-Natal	30
Northern Cape	30
Eastern Cape	43
Western Cape	26
Total	517

### Next-generation amplicon sequencing of *msp1β* and *groEL genes*

Next-generation sequencing (NGS) was used to determine the extent of variation in amplicons of a part of the *msp1β* and *groEL* genes of *A. marginale* and *A. centrale* in, respectively, 40 and 25 known positive field samples from across South Africa. Twenty *A. marginale msp1β* gene sequences from GenBank (accession numbers: M59845, AF110808–AF110810, AF112479, AF112480, AF111195, AF111197, AF221692, AF348137, AF348138, AY841153, KU647713–KU647720) were aligned using CLC Genomics Workbench 7.5.1 (https://www.qiagenbioinformatics.com) and used to design primers Msp1β_F (5′-GAT GAA GCA CCT GAC ACT GGT GAG-3′) and Msp1β_R (5′-CGC GTC GAT TGC TGT GC-3′) in areas conserved in all of these sequences. The primers amplify a 419 bp fragment of the *msp1β* gene spanning the qPCR primer and probe area. The primer pair groEL-ACF and groEL-ACR [20] was used to amplify a 522 bp fragment of the *groEL* gene from both *A. marginale* and *A. centrale*. The primers were modified by adding Illumina-specific adaptor sequences to allow for barcoding of each amplicon and were synthesized at Inqaba Biotechnical Industries (Pretoria, South Africa). The PCRs were performed in a total volume of 25 μl containing 1× Phusion Flash High-Fidelity PCR Master Mix (Thermo Fisher Scientific, Waltham, USA), 1.5 μM of each primer and 2.5 μl genomic DNA (approximately 200 ng). For amplification of the *msp1β* amplicon, the PCR thermal cycling conditions were 98 °C for 10 s, 40 cycles of 98 °C for 5 s, 67 °C for 15 s, 72 °C for 15 s, and a final extension at 72 °C for 1 min. The same cycling conditions were used for amplification of the *groEL* amplicon, except that the annealing temperature was 66 °C. The amplicons were purified using the QIAquick gel extraction kit (Qiagen) according to the manufacturer's instructions.

Plasmid controls were included in determining the *Taq* and sequencing error rate, to distinguish sequence artefacts from real sequence variants [38]. Multiple strains of *A. marginale* are known to be present in South African samples [24, 25], but the relative incidence of different strains in each sample is unknown, and some strains may be present at very low levels. Amplicons were therefore generated from plasmid controls F48a (*A. marginale msp1β*), 9410c (*A. centrale groEL*) and C14c (*A. marginale groEL*) to determine the frequency of sequence artefacts (including *Taq* or sequencing errors) expected in the field samples. The positive control plasmids were generated previously from field samples that were positive for *A. marginale* (F48 and C14) and *A. centrale* (9410) [18].

Resulting amplicons were gel purified, end repaired and Illumina-specific adapter sequences were ligated to each amplicon. Following quantification, the samples were individually indexed, and another purification step was performed. Indexed, adapter-ligated amplicons were then sequenced on Illumina’s MiSeq platform, using a MiSeq v3 (600 cycles) kit (San Diego, California, USA). About 20 MB of data (2 × 300 bp long paired-end reads) were produced for each sample.

Quality filtering was performed on the MiSeq platform, using standard procedures. Only reads that mapped to *A. centrale groEL* 9410c, *A. marginale groEL* C14c and *A. marginale msp1β* F48a reference sequences [18] were incorporated into the subsequent analysis. The sequences were analysed by first merging corresponding Illumina R1 and R2 reads, and only merged sequences were analysed further. Again, the *groEL* and *msp1β* amplicon sequences were mapped to their respective *A. marginale* or *A. centrale* reference sequences. For each set of merged reads, a clustering based on sequence identity was performed. For the *groEL* control plasmid clone 9410c included to determine the frequency of artefacts, the highest proportion of sequences (47.6%) was identical to the 9410c reference sequence. All other sequences (artefacts) were present at an abundance of less than 1.5% each, but collectively made up 52.4% of the sequences. For the *msp1β* plasmid clone F48c, 63.8% of the sequences were identical to the F48c reference sequence, and all other sequences were present at an abundance of less than 1.4%, collectively making up 36.2% of the sequences. Therefore, for the field samples, sequences present at less than 1.5% of the total after cluster analysis were disregarded as *Taq* or sequencing errors. In each cluster, sequences that were present at ≥ 1.5% of the total number of sequences were therefore considered to be true variants and were aligned with published sequences using CLC Genomics Workbench 7.5.1.

### Confirmation of *msp1β* variants by Sanger sequencing

The *msp1β* variants identified by NGS were confirmed by Sanger sequencing in eleven samples. Primers AM456 and AM1164 [19] were used to amplify a 750 bp region of the *msp1β* gene flanking the qPCR target area. The reaction mixture contained 1× Phusion Flash High-Fidelity PCR Master Mix (Thermo Fisher Scientific), 0.5 μM of each primer, 2.5 μl of template DNA (approximately 200 ng) and molecular grade water to a final volume of 25 μl. The PCR thermal cycling conditions were 95 °C for 3 min, 40 cycles of 94 °C for 10 s, 60 °C for 30 s, 72 °C for 30 s, and a final extension at 72 °C for 7 min. The PCR products were purified, quantified and cloned using the CloneJET PCR Cloning Kit (Thermo Fisher Scientific). Recombinant plasmids were screened by colony PCR using vector-specific primers pJET1.2F and pJET1.2R. Plasmid DNA was extracted from recombinants using the High Pure Plasmid Isolation kit (Roche Diagnostics, Basel, Switzerland). Plasmids containing the correct insert were sequenced bidirectionally on an ABI Prism 3100 Genetic Analyzer (Applied Biosystems, Foster City, California, USA) at Inqaba Biotechnical Industries. Sequences were assembled and aligned using CLC Genomics Workbench 7.5.1.

### Quantitative real-time PCR (qPCR) for specific detection of *A. marginale* and *A. centrale*

A duplex qPCR assay with minor modifications for the LightCycler real-time machine (Roche Diagnostics) targeting the *msp1β* gene of *A. marginale* and the *groEL* gene of *A. centrale,* was used to detect *Anaplasma* spp. in genomic DNA samples as previously described [18]. DNA extracted from the *A. centrale* vaccine strain (Onderstepoort Biological Products, Pretoria, South Africa) or field sample 9410 (confirmed to be infected with *A. centrale* by amplification and sequence analysis of the *groEL, msp2* and 16S rRNA genes [18]) were used as positive controls. Field samples C14 or C57 (obtained from cattle in the Mnisi Community area) were used as positive controls for *A. marginale,* and molecular grade water as a negative control. To determine *A. centrale* loads, DNA was extracted from 10-fold serial dilutions of vaccine prepared in uninfected bovine blood. The data were analysed using LightCycler Software version 4.0. (Roche Diagnostics). The linear range of detection and assay efficiency of selected variants were determined as previously described [18].

### Amplification, cloning and sequencing of the *msp1α* gene

The repeat-containing region of the *msp1α* gene was amplified using primers 1733F (5′-TGT GCT TAT GGC AGA CAT TTC C-3′) and 2957R (5′-AAA CCT TGT AGC CCC AAC TTA TCC-3′) [41]. Phusion Flash High-Fidelity PCR Master Mix (Thermo Fisher Scientific) reactions were set up as for *msp1β*. Cycling conditions were 98 °C for 10 s, 30 cycles of 98 °C for 1 s, 69.1 °C for 5 s and 72 °C for 18 s, and a final extension at 72 °C for 1 min. If these PCR conditions failed to generate an amplicon for a sample, the PCR was repeated using the Phusion Flash High-Fidelity PCR Master Mix (Thermo Fisher Scientific), and the cycling conditions reported by [41] except that a pre-PCR denaturation at 94 °C for 3 min and *Taq* activation at 98 °C for 10 s were included. Samples were analysed on a 1.5% agarose gel and those displaying a single, strong band were purified using the Qiagen PCR product purification kit (Qiagen) according to the manufacturers’ instructions. Samples containing multiple PCR products and PCR products that produced mixed sequences were cloned into pJET 1.2 (Thermo Fisher Scientific). Recombinant clones and amplicons were sequenced at Inqaba Biotechnical Industries as described above.

### Analysis of Msp1a repeats to determine strain type

Sequences were assembled and aligned using CLC Genomics Workbench 7.5.1. RepeatAnalyzer [42] was used to identify, curate, map and analyse Msp1a repeats and *A. marginale* strains. New names (UP1 to UP36) were given to novel repeats that were not recognized by RepeatAnalyzer. All South African Msp1a repeats and *msp1α* genotypes ([24, 25] and this study) were pooled and analysed using RepeatAnalyzer, generating diversity metric scores [42]. For comparison, similar analyses on previously published data from Argentina, Brazil, Mexico, the Philippines and USA, were also carried out.

## Results

### Next-generation sequencing of the *groEL* and *msp1β* genes

**Fig. 1:**
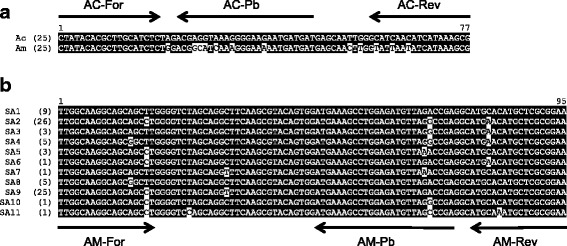
Sequence alignment of groEL and *msp1β* sequences in the qPCR target regions. **a** *A. centrale* (Ac) and *A. marginale* (Am) *groEL* gene sequences obtained in this study. **b** *msp1β* gene sequence variants in the qPCR target region (SA1–SA11) obtained in this study. The number in parentheses after each sequence name indicates the number of samples from which each sequence was obtained. The primer and probe regions are indicated by arrows. Identical nucleotides are shown by white text on a black background while sequence variations are represented by black text on a white background

A total of 39 *A. centrale* and 40 *A. marginale* partial *groEL* sequences (approximately 520 bp in length) were obtained from 25 bovine samples. The *A. centrale groEL* sequences were conserved within the qPCR target region. The *A. marginale groEL* sequences were also conserved and differed from the *A. centrale groEL* sequences at six nucleotide positions in the probe area and three nucleotide positions in the reverse primer region (Fig. 1a). The *A. centrale groEL* sequences were identical to published sequences including those with accession numbers AF414867 (Vaccine strain, South Africa), AF414866 (L strain, South Africa) and ACIS_00394 in the complete genome sequence, CP001759 (Israel strain); while the *A. marginale groEL* sequences were similar to the St. Maries (USA) sequence (AM944 in CP000030). For *msp1β,* 151 different sequences (partial gene sequence; approximately 420 bp in length) were obtained from a total of 183 sequences from 40 samples. Individual samples contained between one and 11 different *msp1β* sequences. Eleven variants (designated as SA1-SA11) were identified in the qPCR target area (Fig. 1b). Single nucleotide polymorphisms (SNPs) were identified at six positions in the primer and probe regions; individual variants contained one to three of these SNPs. Variants SA1, SA2, SA3, SA4, SA5, SA8 and SA9, were identified in multiple samples, while variants SA6, SA7, SA10 and SA11 occurred in only one sample each. The most common variants were SA2 and SA9, identified from 25 samples each. Both of these variants were widespread in South Africa; SA9 occurred in seven provinces, while SA2 was identified in eight provinces. The greatest number of variants (eight) was identified in samples from the Western Cape.

**Fig. 2:**
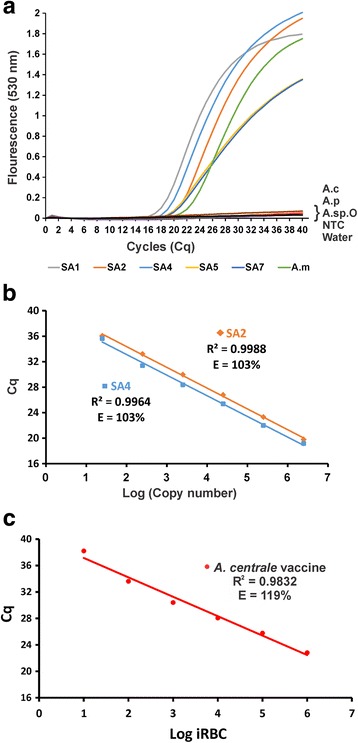
qPCR amplification of *A. marginale msp1β* variants. **a** qPCR amplification of plasmid DNA (2.5 × 10^7^ copies) of *A. marginale msp1β* variants (SA1, SA2, SA4, SA5, SA7). Genomic DNA (gDNA) from sample C14 was used as a positive control for *A. marginale* (A.m) and water as a negative control. gDNA from the *A. centrale* (A.c) vaccine strain, *A. phagocytophilum* (A.p), *Anaplasma* sp. (Omatjenne) (A.spO) and a no temple control (NTC) were included in the analysis. **b** Detection of tenfold serial dilutions (2.5 × 10^7 ^– 2.5 × 10^2^ copies) of plasmid DNA of *A. marginale* variants SA2 and SA4. **c** Detection of tenfold serial dilutions of *A. centrale* vaccine strain (10^6^–10^1^ iRBCs) genomic DNA. *Abbreviations*: C_q_, quantification cycle; R^2^, regression coefficient; E, assay efficiency

Variants SA1, SA2, SA4, SA5 and SA7 were cloned and their sequences confirmed by Sanger sequencing. Plasmid DNA from clones of these five variants could be detected by the qPCR assay (Fig. 2a). qPCR assay efficiency for detection of variant SA1 was evaluated in a previous study [18]. Evaluation of the efficiency of the qPCR assay in detecting the two variants (SA2 and SA4) containing the most differences (3 SNPs) in the primer and probe regions indicated that the SNPs did not have any effect on the efficiency of the assay (Fig. 2b).

### Detection of low *A. centrale* loads in duplex qPCR

Serial dilutions of a known amount of *A. centrale* blood vaccine was used in the duplex qPCR to establish our ability to detect low parasite loads in blood samples (Fig. 2c). We could detect as few as ten infected red blood cells (10 iRBCs) per 20 μl reaction. When working directly from genomic DNA extracted from a blood sample, the efficiency of the qPCR becomes 119%. This apparent increase in efficiency compared to the assay applied to plasmids (E = 103%, Fig. 2b) is likely due to inhibitors co-extracted with the genomic DNA.

### Detection of *A. marginale* and *A. centrale* infections in field samples by the duplex qPCR assay

**Fig. 3:**
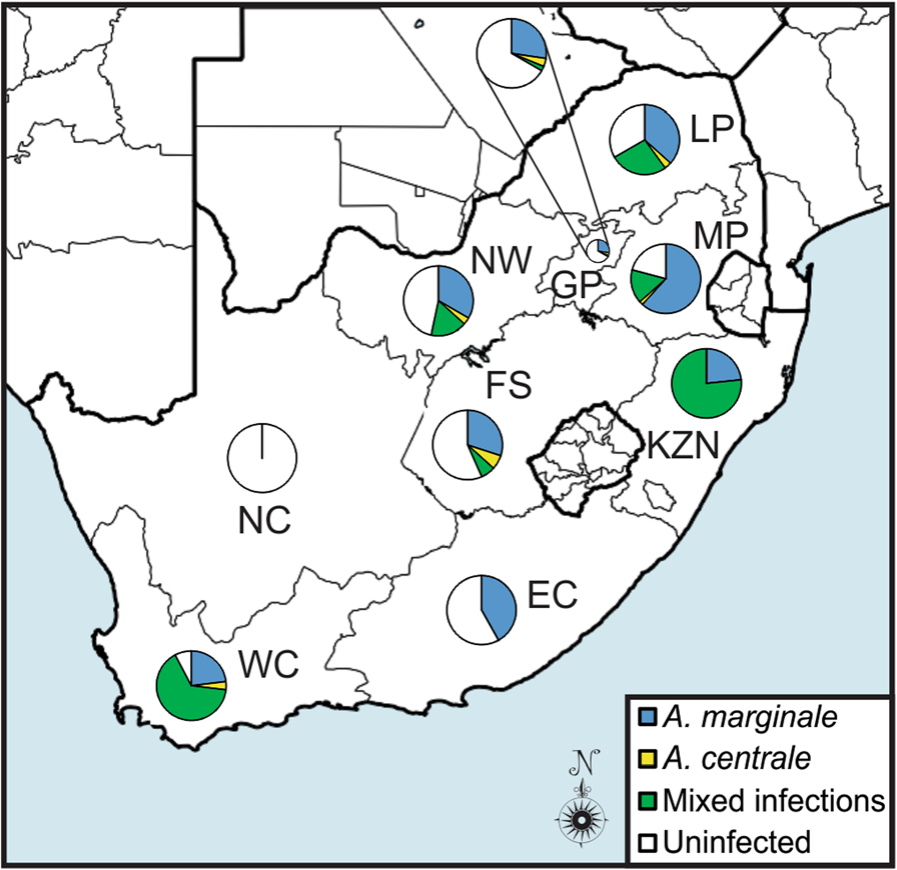
Map of South Africa showing the occurrence of *A. marginale* and *A. centrale* in cattle. DNA extracted from blood samples from cattle from all nine provinces of South Africa were tested for *A. marginale* and *A. centrale* using the duplex qPCR assay [20, 21]. The pie charts indicate the proportion of samples in each province that were positive, negative or which contained mixed infections. *Abbreviations*: GP, Gauteng; EC, Eastern Cape; FS, Free State; KZN, KwaZulu-Natal; LP, Limpopo; MP, Mpumalanga; NC, Northern Cape; NW, North West; WC, Western Cape

FAM fluorescence (530 nm) was generated in *A. marginale*-positive samples and LC-610 (610 nm) signals were generated in *A. centrale*-positive samples. No amplification was detected from the negative control. The qPCR assay detected *A. marginale* and *A. centrale* in 56.8% and 17.2% of the samples (*n* = 517), respectively. Eighty-one (15.3%) samples had mixed infections. *Anaplasma marginale*-positive cattle were identified in all provinces except Northern Cape (Fig. 3). Most of the *A. marginale*-positive samples were identified in KwaZulu-Natal (100%), Western Cape (88.5%) and Mpumalanga (77.4%), while most of the *A. centrale-*positive cattle were from KwaZulu-Natal (76.7%) and Western Cape (69.2%). *Anaplasma centrale* was not identified in samples from the Eastern Cape and Northern Cape.

### *Msp1α* genotyping and sequence analysis of *A. marginale* Msp1a repeats identified in this study

**Fig. 4:**
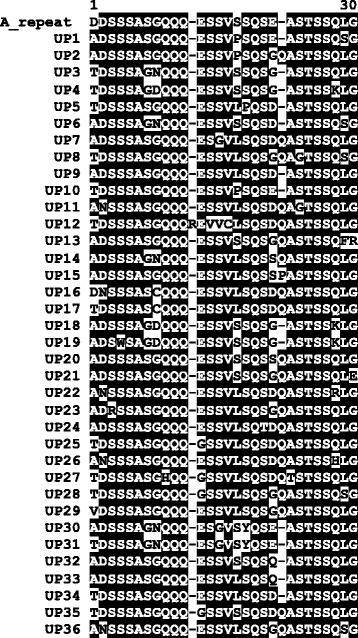
Novel Msp1a sequences repeats found in this study. Thirty-six unique repeats were identified in this study (UP1-UP36) and aligned against the A repeat [28], using the AlignX module of Vector NTI. Identical amino acid residues in the alignment are shown by white text on a black background; variable residues are indicated by black text on a white background

To examine the *A. marginale* strain diversity in the sample set, *msp1α* genotypes were determined in samples that were shown to be *A. marginale*-positive using the duplex qPCR. In our study, a total of 143 genotypes were found from 627 *msp1α* sequences, which were generated from 85 samples from across South Africa. An average of 10.5 samples was analysed per province, and an average of 27.8 genotypes was identified per province. Thirty-six Msp1a repeats that have not previously been reported were found, and these were designated UP1-UP36 (Fig. 4). The novel repeats were 28–29 amino acids in length, except UP12 which was found to have an arginine (R) insertion at position 12, making it the longest repeat at 30 amino acids. Alignment of 234 published repeats shows that Serine (S) residues tend to be highly conserved (data not shown). Interestingly, S residues in the repeat region are thought to be O-glycosylated and to facilitate the adhesion function of the Msp1a protein [43]. The 36 novel repeats (Fig. 4) all contained variations in the previously reported immunodominant and linear B-cell epitope SSAGGQQQESS (positions 4–14), the neutralisation-sensitive B-cell epitope Q/EASTSS (positions 21–26) and the T-cell epitope VSSQSDQASTSSQLG (positions 15–29) [28, 31, 43, 44]. The former B-cell epitope varied at 7 out of 11 positions: 4(S/W), 7 (G/S), 8 (G/N/D/C), 9 (Q/H), 12 (E/G), 13 (S/V) and 14 (S/G/V), while the latter varied at 3 out of 6 positions: 21 (Q/E/G/D/S/P), 22 (A/T) and 23 (S/G). The T-cell epitope had variations at 11 out of 15 positions: 16 (S/L/P), 17 (S/P), 18 (Q/Y), 19 (S/Q/T), 20 (D/G/S), 21 (Q/E/G/D/S/P), 22 (A/T) and 23 (S/G), 27 (Q/K/R/H), 28 (L/F/S), 29 (G/R/E).

### Analysis of Msp1a repeats and *msp1α* genotypes using RepeatAnalyzer

**Fig. 5:**
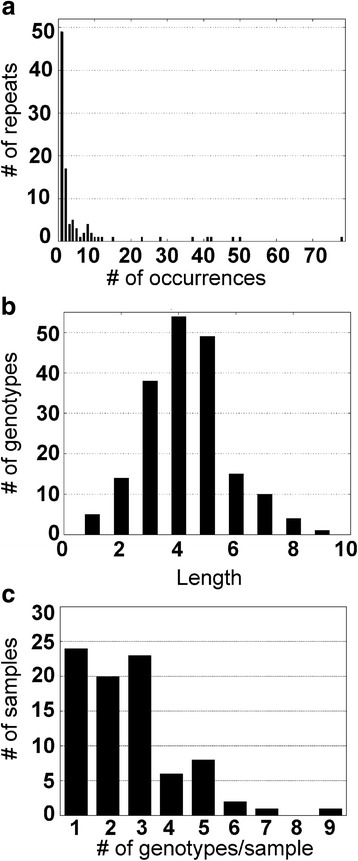
Msp1a repeat and *msp1α* genotype metrics. **a** Frequency distribution of repeats in Msp1a sequences in South Africa generated by RepeatAnalyzer [42]. **b** Genotype-length distribution of Msp1a repeats in South Africa generated by RepeatAnalyzer. **c** The frequency of *A. marginale msp1α* genotypes found per animal in this study (*n* = 85)

For all South African Msp1a data collected to date, from this and previous reports [23, 24], the frequency distribution of Msp1a repeats resembled a power-law distribution (Fig. 5a). Unique repeats (those observed only once in all *A. marginale* genotypes in South Africa) were observed in 48 instances; examples of such repeats are G, 39, 44, T, UP29, 83, 145, and 154. Six Msp1a repeats, 13, 37, 34, 27, 4 and 3, were found to be most common in South Africa, occurring between 37 and 78 times. There was a normal distribution of *msp1α* genotype lengths (Fig. 5b) (μ = 4.26; σ = 1.48), which ranged from one to nine repeats. *Msp1α* genotypes in South Africa most frequently contained four or five repeats; these occurred 53 (27.9%) and 49 (25.8%) times, respectively (Fig. 5b). The frequency of genotypes per sample (Fig. 5c) was found to be positively skewed. A total of 78.8% of the samples contained one (28.2%), two (23.5%) or three (27.1%) genotypes per sample. Four to nine genotypes per sample were also observed, but much less frequently.

**Fig. 6:**
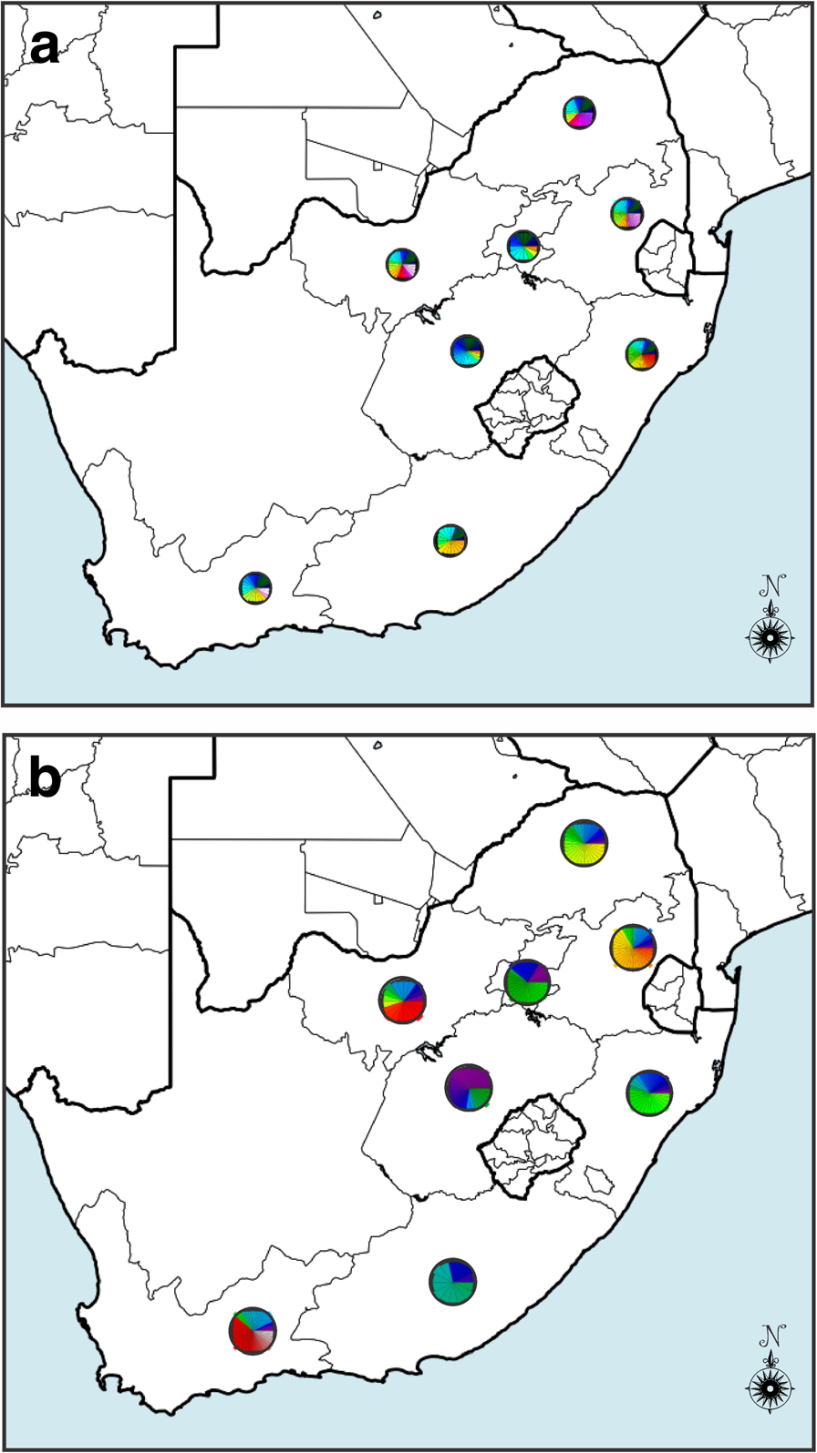
Geographical distribution of Msp1a repeats and strains from *A. marginale* in South Africa. **a** Distribution of 99 Msp1a repeats from *A. marginale* identified in South Africa in this and previous studies. Different colours in each circle represent different repeats, with more colours indicating a higher repeat diversity in each region. **b** Distribution of 190 *A. marginale* strains identified in South Africa in this and previous studies. Different colours in each circle represent different strains, with more colours indicating a higher strain diversity in each region. Results were generated in RepeatAnalyzer [42]

To date, a total of 99 Msp1a repeats (Fig. 6a) have been described in South African *A. marginale* genotypes, 71 (71.7%) of which are unique to the country (Table 2). These repeats are found in a total of 190 *msp1α* genotypes (Fig. 6b)**,** the majority of which appear to be unique to South Africa (Table 3). In general, repeats were fairly evenly distributed around the country (Fig. 6a). The most abundant strains found in this study have been reported previously [24, 25]. These were SW112. 42 43 25 31 (occurring 12 times in five provinces, Mpumalanga, Eastern Cape, Limpopo, KwaZulu-Natal and North West), SW32. 34 13 13 37 (occurring 6 times in five provinces, Western Cape, Mpumalanga, Gauteng, Limpopo, KwaZulu-Natal) and NW-C1-160312. 34 13 3 36 38 (occurring 8 times in five provinces, Mpumalanga, Limpopo, KwaZulu-Natal, Free State). Some *msp1α* genotypes were found in more than one province, while low abundance genotypes which appeared only once in one province were also detected (Fig. 6b).

**Table 2:** Msp1a repeat analysis for different geographical locations, using RepeatAnalyzer

	Location				
	Brazil	USA	Argentina	Mexico	South Africa
Number of unique Msp1a repeats	6	10	12	27	71
Total number of Msp1a repeats	33	22	33	64	99
% unique repeats	18.2	45.5	36.4	42.2	71.7
Other locations with repeats in common	Arg, Mex, SA, USA	Arg, Brz, Mex, SA	Brz, Mex, SA, USA	Arg, Brz, SA, USA	Arg, Brz, Mex, USA
Common repeats appearing in four or more countries	F	F	F	F	F
	M	M	M	M	M
	13	–	13	13	13
	15	–	15	15	15
	18	–	18	18	18
	27	–	27	27	27
	B	B	B	B	–
	C	C	C	C	–
	Q	–	Q	Q	Q
	τ	–	τ	τ	τ

*Abbreviations*: *Arg* Argentina, *Brz* Brazil, *Mex* Mexico, *SA* South Africa, *USA* United States of America

**Table 3:** *Msp1α* genotype analysis for different geographical locations, using RepeatAnalyzer

	Location				
	Brazil	USA	Argentina	Mexico	South Africa
Number of unique *msp1α* genotypes	18	43	15	84	188
Total number of *msp1α* genotypes	23	43	18	89	190
% unique genotypes	78.3	100.0	83.3	94.4	99.0
Other locations with genotypes in common	Mex, Arg, SA	–	Brz, Mex	Brz, Arg	Brz
Genotypes occurring in more than one country	α β β β β γ	–	α β β β β γ	α β β β β γ	–
	–	–	α β β β γ	α β β β γ	–
	α β β γ	–	–	α β β γ	–
	τ 57 13 18	–	–	–	τ 57 13 18
	τ 10 15	–	τ 10 15	τ 10 15	–
	13 27 27^a^	–	–	–	13 27 27^a^
	–	–	–	13 13^a^	–

*Abbreviations*: *Arg* Argentina, *Brz* Brazil, *Mex* Mexico, *SA* South Africa, *USA* United States of America

^a^Also found in the Philippines

Msp1a repeats and *msp1α* genotypes occurring in five selected countries, Brazil, Argentina, Mexico, South Africa and USA, were compared. The percentage of repeats specific to each country (unique repeats) (Table 2) was consistently lower than the percentage of unique genotypes (Table 3). The highest percentage of unique repeats (71.7%) was found in South Africa, while the lowest (18.2%) was in Brazil (Table 2). The most common repeats, which appeared in all of the countries examined, were F and M. Eight other common repeats were found to be present in four of the five countries (Table 2). Although many of the Msp1a repeats identified were found in all five countries examined (an average of 42.8% of Msp1a repeats were unique to each country), very few genotypes were present in more than one country (an average of 91.0% of the *msp1α* genotypes were unique to each country). The highest proportions of unique genotypes were found in USA (100%) and South Africa (99.0%), with Brazil (78.3%) having the lowest observed value (Table 3). More *msp1α* genotypes have been identified in South Africa (190 *msp1α* genotypes) than in any other country, although this likely due to sampling density. Only two genotypes that have previously been identified in other countries were identified in samples from South Africa: (i) τ 57 13 18, found in strain Minas-11 (Minas Gerais, Brazil) [24, 45] was identified in two samples from KwaZulu-Natal; and (ii) 13 27 27, found in strain UFMG-2 (Minas Gerais, Brazil) [24, 45] (also found in the Philippines [39]) was identified in samples from Eastern Cape and Mpumalanga. The genotypes common between South Africa, Brazil and the Philippines represent only 1% of the total number of genotypes described thus far in South Africa.

## Discussion

We have recently shown [18] that the duplex qPCR assay [20] is a more sensitive method of detecting *A. marginale* and *A. centrale* infections in cattle in South Africa than RLB [22] or nPCR [19] assays. We also detected sequence variation in the *msp1β* gene in the target region of one of the nPCR internal primers in South African *A. marginale* strains [18]. The *msp1β* multigene family encodes the Msp1b protein, which has been shown to vary between strains of *A. marginale* [7, 39]. Variation of 0.9–1.4% between Msp1b peptide sequences has been shown, but Msp1b is stable during the bovine and tick stages of the *A. marginale* life-cycle within a given strain [34]. This variation could be detrimental when it is used as a target for detection of the parasite by diagnostic tests such as the *A. marginale*-specific qPCR [21]. Sequence analysis of the *msp1β* gene in the target region of the qPCR assay in the current study indicated that the *msp1β* gene of *A. marginale* from cattle in South Africa was highly variable, many samples had multiple *msp1β* variants (when considering the full-length of the amplicon sequence), and SNPs were present at six nucleotide positions in the primer- and probe-target areas of the qPCR assay. Eleven *msp1β* variants were identified in the qPCR target area.

It has been demonstrated that mismatches located towards the 3′ end of a PCR primer are potentially detrimental to PCR amplification as they can significantly affect annealing of the primer to the template, leading to underestimation of the initial copy number, or even a complete failure of amplification [46]. However, the SNPs identified in this study did not appear to decrease the efficiency of the qPCR assay. The efficiency of the qPCR assay in detection of variants SA2 and SA4 (with the most SNPs) compared well with that of the qPCR assay in detection of SA1 [18] in which there is no variation in the qPCR target region. Nevertheless, the sensitivity of the qPCR assay could still be compromised if there is more variation in the field than we have detected in this study. Moreover, *A. marginale* has been identified from wildlife in South Africa [47], but the sequence variation in the *msp1β* gene in the parasite in these hosts is unknown.

It should be noted that there are two copies of the *msp1β* gene in *A. marginale* [48, 49], and the primers and probe used in the duplex qPCR assay can amplify the target region in both copies. This would explain a large number of samples containing multiple *msp1β* gene variants since many samples contained multiple *A. marginale* strains (as shown by *msp1α* genotyping), and each strain could contain two different *msp1β* copies. The presence of multiple different copies within a sample could increase the likelihood of detecting *A. marginale* since it increases the chance of a single sample containing a variant that can be detected by the qPCR.

The *groEL* gene of prokaryotes, homologous to the heat-shock protein gene in eukaryotes [50], is highly conserved but contains variable regions that can be useful in differentiating closely related organisms [51, 52]. In contrast to the *A. marginale msp1β* gene, the *groEL* genes of *A. centrale* and *A. marginale* were highly conserved in the target region of the qPCR assay, although SNPs in other regions of this gene were identified. Since the sequence differences targeted by the qPCR primers and probes were highly conserved in all *A. centrale* and *A. marginale groEL* sequences examined, the *groEL* gene is, therefore, a good marker for the detection of *A. centrale* infections in cattle in South Africa. However, in a recent study on the occurrence of tick-borne infections in cattle samples from Uganda [53], RLB assay detected more *A. centrale* infections than the qPCR assay, indicating the possibility of *groEL* gene variants which cannot be detected by the qPCR assay. This highlights the necessity for testing the assay in each region in which it is to be deployed. Further, the detection limits are shown to be approximately ten iRBC/reaction; although this is not being used as a quantitative assay, this can be used as a guideline for field sample detection.

Only two natural isolates of *A. centrale* have been made in South Africa, the original isolate made by Theiler [54] that is used in the blood vaccine, and a second isolate that was made when unfed adult *Rhipicephalus simus* ticks collected in the Louis Trichardt district of the Northern Transvaal (now Limpopo) were fed on a splenectomized ox and an *A. centrale* infection was transmitted [17, 55]. Very little work has been done on this strain of *A. centrale* although it has been shown to have a close identity to Theiler’s *A. centrale* vaccine strain by phylogenetic analysis of the 16S rRNA and *groEL* genes [56]. The *groEL* sequence from this strain (accession no. AF414866) [56] was included in our analysis, and, as with all the other *A. centrale groEL* sequences analysed, there was no variation in the qPCR target region. It is possible that some of the *A. centrale* infections detected in field samples in this study were due to this strain.

Our results indicated that *A. marginale* is widespread in cattle in eight of the nine provinces of South Africa. As expected, high percentages (> 70%) of *A. marginale*-positive samples were identified in KwaZulu-Natal, Western Cape and Mpumalanga, since endemic stability is established in these regions. No *A. marginale* infections were detected in cattle from the Northern Cape; this is consistent with the results from a recent study [25] and was expected since the tick vectors do not occur in this province. Interestingly, *A. centrale* was also detected in the cattle, although none of them was known to have been vaccinated, and mixed infections of *A. marginale* and *A. centrale* were common. A high percentage of cattle from KwaZulu-Natal and Western Cape were positive for *A. centrale,* suggesting that this organism is more common in the southern provinces of South Africa. However, it was not detected in cattle samples from the Eastern Cape, but this may have been an artefact of the sampling (43 samples were collected from five farms in two of 39 local municipalities, representing only 3.8% of the area of the Eastern Cape); more samples should, therefore, be sourced from this province to increase confidence in this result. This is the first comprehensive study on the occurrence of *A. centrale* in cattle in all nine provinces of South Africa using a nucleic acid-based method, although we recently reported on the occurrence of this species in cattle in Bergville, KwaZulu-Natal province, South Africa [47]. Mixed infections of *A. centrale* and *A. marginale* have been reported in cattle and wildlife in South Africa [47] and in cattle elsewhere [20, 53, 57]. Although multiplex qPCR assays are recommended for detecting tick-borne pathogens, competitive PCR suppression may occur if infection levels are similar between two or more target species, or are higher in one species/target [58]. This can affect assay sensitivity as has been reported with multiple infections of *T. parva*, *Theileria* sp. (buffalo) and *Theileria* sp. (bougasvlei) in buffalo [58]. Decaro et al. [20] partly addressed this problem by increasing the concentration of the *A. centrale* primers to increase the chance of detecting this pathogen in mixed infections.

*Msp1α* genotyping revealed that most qPCR-positive cattle (71.8% of samples) in this study were found to be infected with multiple *A. marginale* strains. This is expected in endemic areas and has been reported in previous studies in the USA and the Philippines [36, 39]. Although up to nine *msp1α* genotypes were found per animal, the most abundant genotypes were one to three genotypes per sample. Competition for limited niches or resources in a single host is likely to increase with increasing number of genotypes and may explain the lower numbers of genotypes per animal. Moreover, in South Africa, oxytetracycline and imidocarb are bought over-the-counter by farmers without the need for a veterinary prescription, and these drugs are commonly used to treat babesiosis, heartwater and anaplasmosis, the most common tick-borne diseases in South Africa [3]. Therefore, treatment regimens used by farmers and veterinarians, which have been shown to reduce infection in animals [2, 59], combined with host immunity [2], may play an important role in maintaining lower numbers of genotypes per animal.

*Msp1α* genotype has been shown to be a surrogate indicator for strain antigenicity, with strains with different *msp1α* genotypes having different *msp2* repertoires [23]. Futse et al. [60] demonstrated that a single unique *msp2* allele was sufficient for a strain to establish superinfection in the face of robust immunity to a primary infecting strain. Our results may suggest superinfection by genomically distinct *A. marginale* strains, which is thought to be uncommon in the temperate regions of the world but occurs more frequently in the tropics [37, 38, 40]. However, superinfection cannot be proven to have occurred in our samples as the infection progress was not monitored in the animals over time, only assessed at one static time point.

Our results demonstrate the importance of cloning all *msp1α* PCR products when genotyping *A. marginale* to detect multiple infections per animal. Previous studies have focused on samples with only a single detectable band, and have only sequenced one product. To fully explore the diversity of genotypes in a given sample, an investigator must analyse all *msp1α* amplicons obtained. The detection of 36 low abundance, previously undescribed *A. marginale* repeats in this study, emphasizes this point. It should be noted, however, that since *msp1α* is a repetitive sequence, errors in PCR are possible if amplification halts and one repeat primes amplification on another, leading to genotypes with extra repeats. Such a situation may have occurred in up to six samples (7.1%) in this study. Errors may also occur due to *Taq* polymerase slippage early in the PCR, resulting in over- or under-representation of certain repeats. Other error sources may be due to low DNA concentration or poor sample quality, which may arise from improper storage or repeated cycles of freezing and thawing of blood samples (reviewed in [61]).

Worldwide, over 250 highly variable Msp1a repeats have been detected to date [8, 31, 42]. The amino acid sequences of the B- and T-cell epitopes that have previously been identified and shown to be necessary to elicit a protective immune response by Msp1a [28, 31, 32, 43, 44], were found to be variable in the novel Msp1a repeats described in this study, and this variation almost certainly has an effect on the overall epitope structure. Such variations should, therefore, be considered when testing Msp1a as a protective antigen. Serine residues at positions 4 and 25, however, were found to be highly conserved; these residues are thought to be important for O-glycosylation and the adhesion function of the protein, which is essential for transmission of *A. marginale* [43].

We found that 28 out of the 99 (22.3%) Msp1a repeats identified in South Africa are also found in strains in other countries, but this does not translate to many shared genotypes, with only two genotypes out of 190 (approximately 1%) found in common between South Africa and Brazil, and the Philippines. This result is in concordance with a recent study analysing global repeat and strain distribution [31]. These data may suggest that new repeats arise independently in different geographical regions, resulting in the emergence of novel genotypes, which arise from new repeat combinations. Interestingly, one of the two genotypes that was found to be common between South Africa and Brazil (τ 57 13 18), had a repeat structure which differed by one repeat from one of the world’s most common genotypes, τ 22 13 18, which has been detected seven times in Argentina and Mexico [31] (repeats 57 and 22 differ by eight amino acids). Although the low prevalence of genotypes common between South Africa and the rest of the world may be due to restricted cattle movements, it could also be due to a lack of *A. marginale* genotyping efforts in other parts of Africa and some regions of the world.

We have identified a large number of diverse Msp1a repeats which are fairly evenly dispersed in South Africa. A large proportion of these Msp1a repeats and *msp1α* genotypes are found only in South Africa. High repeat and genotypic diversity, and an even dispersion of repeats are expected in situations where the number of region-specific repeats and genotypes is high [42, 47], which is evident in the South African data. These data may suggest that repeats (and their associated genotypes) are circulating within the country as a whole, a process which may be driven by cattle movement between the high prevalence endemic areas and the presence of tick vectors of *A. marginale* to propagate the pathogen. In fact, more than one genotype was found to be common between three to five provinces, which provides evidence of ongoing movement of cattle between provinces within South Africa. Both artificial and natural selection factors such as the presence and control of competent tick vectors, host immunity and chemotherapy treatment, are strong determinants of *A. marginale* repeat and genotype composition in different areas. This study demonstrates a high genetic variability of the *A. marginale* population in South Africa, which is an important factor to consider in formulating future vaccine design strategies.

## Conclusions

Both *A. marginale* and *A. centrale* are prevalent in South Africa. *Anaplasma centrale* was detected in cattle despite the lack of vaccination with this organism, suggesting that there is a natural transmission cycle of *A. centrale* in South Africa. A total of 190 different *msp1α* genotypes of *A. marginale* have been detected in South Africa, indicating a diversity of genotypes that must be taken into account when developing a vaccine.

## Abbreviations

C_q_: Quantification cycle
DNA: Deoxyribonucleic acid
E: Assay efficiency
EDTA: Ethylenediaminetetraacetic acid
gDNA: Genomic DNA
iRBCs: Infected red blood cells
Msp1a: Major surface protein 1a
*msp1α*: Gene encoding major surface protein 1a
*msp1β*: Gene encoding major surface protein 1b
NGS: Next-generation sequencing
nPCR: Nested polymerase chain reaction
PCR: Polymerase chain reaction
qPCR: Quantitative real-time polymerase chain reaction
R^2^: Regression coefficient
RLB: Reverse line blot
rRNA: Ribosomal ribonucleic acid
SNP: Single nucleotide polymorphism
